# Red sacaca essential oil-loaded nanostructured lipid carriers optimized by factorial design: cytotoxicity and cellular reactive oxygen species levels

**DOI:** 10.3389/fphar.2023.1176629

**Published:** 2023-10-11

**Authors:** Sofia Santos Donaire Chura, Kathelen Anne Sudo Memória, Amanda Tibães Lopes, Franciele Maria Pelissari, João Vinícios Wirbitzki Da Silveira, Jaqueline de Araújo Bezerra, Francisco Celio Maia Chaves, Ana Paula Rodrigues, Jerusa Araújo Quintão Arantes Faria, Guilherme Carneiro

**Affiliations:** ^1^ Department of Pharmacy, Faculty of Biological and Health Sciences, Federal University of Jequitinhonha and Mucuri Valleys, Diamantina, Brazil; ^2^ Postgraduation Program in Basic and Applied Immunology, Federal University of Amazonas, Manaus, Brazil; ^3^ Institute of Science and Technology, Federal University of Jequitinhonha and Mucuri Valleys, Diamantina, Brazil; ^4^ Federal Institute of Education, Science and Technology of Amazonas (IFAM), IFAM Analytical Center, Manaus Centro Campus, Manaus, Brazil; ^5^ Embrapa Western Amazon, Manaus, Brazil

**Keywords:** amazon plants, rotational central composite design, *Croton cajucara* Benth., nanotechnology, plant metabolites

## Abstract

Amazonian flora includes several species with the potential to develop pharmaceutical and biotechnological products. The essential oils from Amazonian species possess some biological properties, such as antioxidant, antibacterial, and cytotoxic activities. The essential oil of red sacaca (RSO), *Croton cajucara* Benth., contains metabolites characterized by antioxidant and anti-inflammatory activities. Nanostructured lipid carriers (NLC) are an advantageous alternative for the effective delivery of drugs because they can solubilize lipophilic actives and reduce their cytotoxicity. This study aimed to optimize the synthesis of RSO-loaded nanostructured lipid carriers (NLC-RSO) using a 2^3^ factorial design and investigate their antioxidant and cytotoxic effects. The red sacaca essential oil (RSO) metabolite profile was characterized using gas chromatography coupled with a mass spectrometer (GC-MS), identifying 33 metabolites, with linalool and 7-hydroxy-calamenene as the major ones, as reported in the literature. The optimized NLC-RSO formulation had a particle size less than 100 nm and a polydispersity index lower than 0.25. After characterizing NLC-RSO using Fourier-transform infrared spectroscopy, powder X-ray diffraction, zeta potential, moisture content, and wettability, *in vitro* cytotoxicity were performed in A549 and BEAS-2B cell lines using the resazurin metabolism assay. The data indicated a lower IC50 for RSO than for NLC-RSOs in both cell lines. Furthermore, low cytotoxicity of blank nanoparticles (blank NP) and medium chain triglycerides-loaded nanostructured lipid carriers (NLC-MCT) towards both pulmonary cell lines was noted. At a concentration of 50–100 μg/mL, free RSO exhibited higher cytotoxicity than NLC-RSO, demonstrating the protective effect of this lipid carrier in reducing cytotoxicity during metabolite delivery. Similarly, free RSO showed higher 2,2-diphenyl-1-picrylhydrazyl (DPPH) radical scavenging than NLC-RSO, also indicating this protective effect. The 2′,7′-dichlorofluorescein diacetate (DCFH-DA) intracellular reactive oxygen species (ROS) level assay did not show differences between the treatments at higher but non-cytotoxic dosages. Taken together, our results suggest that NLC-RSOs are potential RSO delivery systems for applications related to cancer treatment.

## 1 Introduction

The Amazon represents a large part of the Brazilian biome, whose biodiversity is mostly unknown, and drives the search for developing new drugs from plants. The Amazon has a vast plant diversity, from which it is possible to extract essential oils, which, from their secondary metabolites, produce derivative smells and flavors, in addition to presenting a diverse range of metabolites of pharmacological interest (Ferreira et al., 2022). One of the most prevalent Amazonian plant species that provides essential oils is *Croton cajucara* Benth. (Euphorbiaceae), a 6–10 m tall tree found in the central and eastern regions of the Amazon rainforest. Two morphotypes of this species have been described: the white sacaca with light green leaves, and the red sacaca, with dark green leaves (Chaves et al., 2006; Azevedo et al., 2021). Sacaca leaves and bark are routinely commercialized in open markets as oils, capsules, and pills for use as analgesics and hepatoprotective agents, as well as for the treatment of diabetes, diarrhea, stomachaches, fever, hepatitis, and malaria (Azevedo et al., 2021).

Phytochemical studies have indicated that terpenes are the main metabolites of red sacaca essential oil (RSO), including 7-hydroxy-calamenene (35.4%) and linalool (11.8%) (Chaves et al., 2006; Rodrigues et al., 2013; Azevedo et al., 2021; Subramaniyan et al., 2022). Some studies have shown the potential biological activities associated with RSO, including antileishmanial, antimicrobial, antiulcerogenic, anticancer, and antioxidant activities. Against *Leishmania chagasi*, RSO showed an *in vitro* minimum inhibitory concentration of 250 μg/mL, with no significant toxicity to mouse peritoneal macrophages (Rodrigues et al., 2013; Da Silva et al., 2018). High antimicrobial activity was observed from 7-hydroxy-calamenene-rich RSO against methicillin-resistant *Staphylococcus aureus*, *Mycobacterium tuberculosis*, *Mycobacterium smegmatis*, *Rhizopusoryzae*, and *Mucor circinelloides*. A 7-hydroxy-calamenene-rich RSO nanoemulsion proved to be effective against zygomycete strains, especially *Mucor ramosissimus* and *Candida albicans* (Azevedo et al., 2016; Azevedo et al., 2021). Antiulcerogenic and antineoplastic properties have also been attributed to sacaca, which are associated with trans-dehydrocrotonin, a norditerpene found in the bark (Freire et al., 2003; De Carvalho et al., 2022).

Although essential oils present strong potential for use in the pharmaceutical industry, they usually have some limiting characteristics, such as the susceptibility to oxidation and degradation under environmental conditions, low aqueous solubility, and low bioavailability at the site of action (Santos et al., 2017; Rodrigues et al., 2018; Lammari et al., 2020). Thus, the incorporation of oils and lipophilic compounds into lipid nanoparticles has been searched in the pharmaceutical industry to protect the bioactive compounds and overcome such limitations, while using nanostructured components with low risk of acute or chronic toxicity. Nanostructured lipid carriers (NLC) are lipid nanoparticles composed of solid lipids (such as long-chain triglycerides and fatty acids), liquid lipids (oils, such as RSO and other vegetable oils) and the surfactant system (Chu et al., 2017; Salgado et al., 2018; Zanetti et al., 2019). Optimal sized lipid nanoparticles can be obtained from the proper combination of these components, yielding improved properties for the incorporated bioactive, such as increased cellular uptake and potential for passive and active targeting to the regions of interest in the human organism (Bayón et al., 2019; Borges et al., 2020).

Therefore, the main goal of this study was to develop RSO-loaded lipid nanoparticles through a 2^3^ factorial design to optimize its composition, in order to obtain nanoparticles of reduced size that can protect the biological actions of RSO, i.e., maintaining or improving its bioactive effectiveness, without increasing toxicity. It is expected that these novel RSO lipid nanoparticles can be a technological advancement in the rational use of this Amazonian oil, which may lead to increased bioavailability, whose effect may last longer after use.

## 2 Materials and methods

### 2.1 Materials

Red sacaca leaves were collected from the medicinal plants sector of Embrapa Amazônia Ocidental, (localization 03°06′23,04″S e 60°01′35,14″W) in Manaus, Amazonas, Brazil. The access to the botanical material was registered on the National System of Management of Genetic Heritage and Associated Traditional Knowledge (SISGEN) under the number AD070A3. The red sacaca essential oil was obtained by hydrodistillation in a modified Clevenger apparatus (LCR Cientifica; Cosmopolis, Brasil) for 4 h, from plants collected between 08:00–09:00 a.m., as reported previously (Azevedo et al., 2016; Silva et al., 2022).

Compritol^®^ 888 ATO was kindly supplied by Gattefossé (Lyon, France), Super refined Tween^®^ 80 (polysorbate 80) was kindly provided by Croda Inc. (Edison, WI, United States), and soybean lecithin was obtained from Cargill (Krefeld, Germany). Butyl hydroxytoluene (BHT) was acquired from Sulfal (Belo Horizonte, Brazil). The reagents 2,2-diphenyl-1-picrylhydrazyl (DPPH∙), 7-hydroxy-3H-phenoxazin-3-one 10-oxide (resazurin) and 2ʹ,7ʹ-diclorofluorescein diacetate (DCFDA) were acquired from Sigma-Aldrich (St. Louis, MO, United States). All the other reagents were of analytical grade.

### 2.2 Experimental design

To develop this novel formulation of lipid nanoparticles with RSO, the first step was to identify the factors that could influence critical quality attributes. A 2^3^ central composite rotational design was then performed with six repetitions at the central point for 20 experiments. The independent variables, the proportion of RSO in the oily phase (X_1_), total concentration of lipids in the dispersion (X_2_), and total concentration of surfactants in the dispersion (X_3_), were analyzed at three equidistant levels (coded as −1, 0, and +1), with two axial points (−1.68 and +1.68). Two response variables were measured: the particle size (Y_1_) and polydispersity index (Y_2_).

The experimental design and coded and real values of the independent variables are listed in Table 1. The ranges of the independent variables were defined in previous experiments. A second-order model was used to adjust the response variables.

$$Y=\beta_{0}+\sum \beta_{i}X_{i}+\sum \beta_{i}^{2}X_{i}^{2}+\sum \beta_{ij}X_{i}X_{j}$$

where Y is the dependent variable, X_i_ and X_j_ are the coded independent variables, β_0_ is the constant, β_i_ is the linear coefficient, β_i_^2^ is the quadratic coefficient, and β_ij_ is the interaction coefficient.

**TABLE 1:** Central composite design matrix and characterization of the obtained nanoparticles by particle size and PDI as the response variables.

Test	Independent variables			Particle size (nm)	PDI
	X_1_	X_2_	X_3_		
1	−1.00 (18.1)	−1.00 (3.8)	−1.00 (1.8)	131 ± 1	0.25 ± 0.01
2	−1.00 (18.1)	−1.00 (3.8)	1.00 (4.2)	69 ± 1	0.20 ± 0.00
3	−1.00 (18.1)	1.00 (12.2)	−1.00 (1.8)	795 ± 46	0.66 ± 0.03
4	−1.00 (18.1)	1.00 (12.2)	1.00 (4.2)	265 ± 8	0.37 ± 0.05
5	1.00 (41.9)	−1.00 (3.8)	−1.00 (1.8)	127 ± 2	0.23 ± 0.01
6	1.00 (41.9)	−1.00 (3.8)	1.00 (4.2)	68 ± 1	0.23 ± 0.01
**7**	1.00 (41.9)	1.00 (12.2)	−1.00 (1.8)	367 ± 19	0.38 ± 0.06
8	1.00 (41,9)	1.00 (12.2)	1.00 (4.2)	197 ± 2	0.25 ± 0.01
9	−1.68 (10)	0.00 (8)	0.00 (3)	197 ± 8	0.40 ± 0.01
10	1.68 (50)	0.00 (8)	0.00 (3)	150 ± 1	0.22 ± 0.01
11	0.00 (30)	−1.68 (1)	0.00 (3)	38 ± 1	0.25 ± 0.01
12	0.00 (30)	1.68 (15)	0.00 (3)	442 ± 39	0.86 ± 0.03
13	0.00 (30)	0.00 (8)	−1.68 (1)	612 ± 59	1.00 ± 0.00
14	0.00 (30)	0.00 (8)	1.68 (5)	133 ± 6	0.26 ± 0.05
15	0.00 (30)	0.00 (8)	0.00 (3)	186 ± 17	0.35 ± 0.08
16	0.00 (30)	0.00 (8)	0.00 (3)	193 ± 20	0.35 ± 0.08
17	0.00 (30)	0.00 (8)	0.00 (3)	218 ± 6	0.41 ± 0.01
18	0.00 (30)	0.00 (8)	0.00 (3)	181 ± 4	0.30 ± 0.05
19	0.00 (30)	0.00 (8)	0.00 (3)	186 ± 6	0.31 ± 0.04
20	0.00 (30)	0.00 (8)	0.00 (3)	186 ± 2	0.27 ± 0.01

X_1_ = Proportion of RSO, in the oily phase (% w/w); X_2_ = Total concentration of lipids (% w/v); X_3_ = total concentration of surfactants (% w/v).

Statistical analysis of the experimental data and response surface methodology were performed using Statistica 7.0 software (StatSoft Inc., Oklahoma, United States). The optimal values for the formulation composition were obtained by the desirability function, an analysis of multiple responses, as proposed by Derringer and Suich (1980).

To determine whether the fitted equations are significant, the R^2^ value must be equal or greater than 0.70 (Santos et al., 2021).

### 2.3 Preparation of nanoparticles

Red sacaca essential oil-loaded nanostructured lipid carriers (NLC-RSO) were prepared by hot-melt homogenization using an emulsification-ultrasound, as previously described by our group (Marcial et al., 2017; Silva et al., 2023). Briefly, lipids and surfactants were mixed and heated to 85°C for complete melting and homogenization. Subsequently, RSO was added to this mixture, homogenized, and dispersed in 10 mL of previously heated water at the same temperature by stirring for 2 min. The resulting emulsion was immediately homogenized using a high-intensity ultrasound probe (Q55 sonicator, Qsonica, Newton, United States) for 10 min at 40% amplitude. The hot emulsified mixture was then cooled to room temperature (near 25°C) for nanoparticle formation. Blank nanoparticles (blank NP) were prepared with all components, except for RSO, using the same procedure. Medium-chain triglycerides (MCT) were used in the same proportion as RSO to generate the nanostructured lipid carriers containing medium-chain triglycerides (NLC-MCT).

For the powder characterization, the water dispersion containing NLC-RSO was frozen in a nitrogen bath for 5 min and lyophilized in a Labconco FreeZone 4.5 L freeze-dryer (Kansas, EUA), with the condenser system operating at 50°C and vacuum pressure of 0.37 mbar for 24 h.

### 2.4 Determination of particle size and polydispersity index

The hydrodynamic diameter and the polydispersity index (PDI) were determined by dynamic light scattering (DLS) at 25°C and angle of 173°, with a Zetasizer Nano ZS (Malvern Instruments; Worcestershire, England). The formulations were ten-fold diluted in water before analysis, and all determinations were performed in triplicate.

### 2.5 Characterization of the NLC-RSO

#### 2.5.1 Zeta potential

Zeta potential (ZP) was determined by DLS and electrophoretic mobility with a Zetasizer Nano ZS (Malvern Instruments; Worcestershire, England), at 25°C and detection at an angle of 173°. The formulations were ten-fold diluted in water before analysis and determinations were performed in triplicate.

#### 2.5.2 Fourier-transform infrared spectroscopy (FTIR)

The FTIR spectra of the pure components and lyophilized nanoparticles (NLC-RSO and blank NP) were obtained using an FTIR Varian 640-IR spectrophotometer (Palo Alto, United States) equipped with an attenuated total reflectance mode accessory (ATR, Pike Technologies, model GladiATR). Measurements were performed between 650 and 4,000 cm^−1^, with resolution of 4 cm^−1^ and 32 scan accumulations. The thermal stability of RSO was also analyzed by FTIR after thermal treatment at 85°C for 10 min to verify the possible degradation during nanoparticle preparation.

#### 2.5.3 Powder X-ray diffraction (XRD)

XRD data of lyophilized NLC-RSO and pure solid components were collected on the XRD-6000 diffractometer (Shimadzu, Kyoto, Japan) at room temperature, under 40 kV, 30 mA, using CuKa radiation (λ = 1.54056 Å). The sample was scanned over the angular range of 10°–50° (2θ) with a scan speed of 0.06° 2θ·s^−1^ (3.6°/minutes).

#### 2.5.4 Color parameters

Color parameters were determined using a Konica Minolta CM-5 colorimeter with a CIE Standard Illuminant D65, a 10° observer, and specular reflectance, based on the CIE Lab color spaces (Barros et al., 2014; VIEIRA et al., 2019). The color parameters, lightness L^*^, and chromas a^*^ and b^*^, were determined by direct analysis using a colorimeter. The total color difference (ΔE) was calculated using the equation:

$$∆E^{*}=\sqrt{{\left(L^{*}-L_{0}^{*}\right)}^{2}+{\left(a^{*}-a_{0}^{*}\right)}^{2}+{\left(b^{*}-b_{0}^{*}\right)}^{2}}$$

where L^*^ is the luminosity coordinate, a^*^ is the color coordinate between red and green, and b^*^ is the coordinate between yellow and blue. Comparisons were performed using the values of L_0_^*^, a_0_^*^, and b_0_^*^ as the chromatic coordinates for RSO and Compritol.

#### 2.5.5 Moisture content and wettability

Moisture content was determined using a gravimetric method. The lyophilized NLC samples were heated at 105°C until reaching constant weight (AOAC, 2005).

Wettability was determined as the time necessary for the disappearance of 50 mg of sample powder (NLC-RSO) from the surface of 10 mL of distilled water at 25°C without stirring (Fuchs et al., 2006).

### 2.6 Characterization of RSO

#### 2.6.1 RSO metabolites

The metabolites present in RSO were analyzed by gas chromatography coupled with mass spectrometry (GC-MS) according to a previously published report (Mar et al., 2018). A ShimadzuGCMS-QP2010 (Shimadzu Corporation, Kyoto, Japan) instrument with conditions of injector operation at 250°C and detector at 290°C was used in all analyses. Helium was used as carrier gas at a flow rate of 1.0 mL/min, the column was heated from 60°C to 250°C at a rate of 3°C/min, and the split ratio was 1:50. Identification of the isolated metabolites was established from their GC retention index using a C7–C30 n-alkanes homologous series, whose Arithmetic Index (AI) were calculated using the van Den Dool and Kratz (1963) equation.

#### 2.6.2 Radical scavenging measurements

The radical scavenging (RS) measurements of RSO and NLC-RSO was evaluated by the discoloration of DPPH radicals in an acetone solution (Bhatnagar et al., 2009) at 515 nm. Briefly, test tubes were filled with 2 mL of the sample and 2 mL of 60 μM DPPH∙ solution, vortexed and incubated at 25°C protected from the light. After 30 min, the absorbance of the samples (As) or control (DPPH∙ solution; Ac) was determined on a UV-visible spectrophotometer (BEL photonics SP 2000 UV, Piracicaba, Brazil). The RSO concentration range analyzed was 0.78–25 mg/mL. Results were expressed as RS towards DPPH∙ radicals, calculated using the following equation:

$$\%RS=\frac{Ac-As}{Ac}x100$$

The BHT equivalent antioxidant capacity (mg/g of oil) was determined from an analytical curve for BHT in the concentration range of 7.32–50 μg/mL. For comparative analysis, the NLC-RSO samples were diluted to 6.25 mg/mL.

### 2.7 *In vitro* cytotoxicity studies

#### 2.7.1 Cell cultures

The cell lines used were A549 (ATCC^®^ CCL-185™), human lung carcinoma, and BEAS-2B (ATCC^®^ CRL-9609™), normal lung epithelial cells. The cultures were maintained in Dulbecco’s Modified Eagle Medium: Nutrient Mixture 12 (DMEM/F12) supplemented with 1.2 g/L sodium bicarbonate, 10% fetal bovine serum (FBS), 100 units/mL penicillin and 100 μg/mL streptomycin (Thermo Fisher Scientific, Waltham, MA, United States).

The culture medium was filtered through a 0.22 µm polyvinylidene difluoride membrane (Sigma-Aldrich) Cells were grown in flasks in a 37°C, 5% CO_2_ humidified incubator. The culture medium was replaced every 2 days. The cell subcultures were performed when the monolayers reached an approximate confluence of 70%–80%.

#### 2.7.2 Cell viability studies

A resazurin reagent metabolization assay was used to evaluate cell viability and obtain the IC_50_ profile of NLC-RSO. Both cell lines were seeded at a cell density of 1 × 10^4^ cells per well in a standard 96-well tissue culture plate. After 24 h, the cells were washed with phosphate-buffered saline, and different concentrations of NLC-RSO, blank NP, NLC-MCT, MCT, and RSO were applied. After the incubation of the cell cultures with the treatments for 24 h in the 96-well plates, 3 mM resazurin reagent was added to each well, and the plate was further incubated for 3 h under 5% CO_2_ at 37°C. The absorbance of each sample was measured at 570 and 590 nm using Chameleon multi-label plate reader (Hidex Personal Life Science, Hidex Oy, Finland). Results were expressed as percentage of viability compared to the untreated cells.

### 2.8 Quantification of cellular reactive oxygen species levels

For this study, A549 and BEAS-2B cells were plated at 1 × 10^4^ and 2 × 10^4^ cells/well, respectively, in 96-well plates. After the adherence period (24 h), the cell lines were treated with 25 μg/mL of each treatment for 24 h, then washed with 1X Hank’s balanced salt solution (HBSS). All groups received 25 µM of the fluorescent probe 2′,7′-dichlorofluorescein diacetate (DCFDA) in 1X HBSS solution for 45 min. After this period, the probe was washed with 1X HBSS and a part of the groups was subjected to oxidative stress using 50 µM of hydrogen peroxide (H_2_O_2_) in 1 X HBSS solution, while the other group of cells received only the 1 X HBSS solution. Fluorescence readings (excitation at 488 nm and emission at 535 nm) were performed after 1 h. For this test, the antioxidant quercetin at a concentration of 200 µM was used as a positive control group (Tang et al., 2019). Results were expressed as percentage fluorescence (DCFDA) relative to control group (non-treated cells).

### 2.9 Statistical analyses

Data were obtained from at least three independent experiments and expressed as mean ± standard deviation. The optimal values for the formulation composition were obtained using the desirability function and analysis of multiple responses, as proposed by Derringer and Suich (1980), using Statistica 7.0 software (StatSoft Inc., Oklahoma, United States). Viability and antioxidant statistical analyses were performed using the GraphPad Prism 8 software (La Jolla, CA, United States). To obtain the IC_50_ values, a nonlinear regression analysis was performed. To evaluate the significance of the differences observed between the studied groups, a one-way analysis of variance (ANOVA) was conducted and, when applicable, the mean comparison test (Tukey’s test). All data obtained from the factorial experiments were subjected to statistical analysis at 90% confidence level for fitting to the second order polynomial equation.

## 3 Results and discussion

### 3.1 Influence of the formulation parameters on the particle size and PDI of NLC-RSO

After preparation, all NLC-RSO formulations in the experimental design were fluid and transparent, with a reddish appearance. Their average sizes ranged between 38 and 795 nm, indicating the formation of nanostructures in all composition combinations. The PDI values ranged between 0.20 and 1.00 (Table 1).

All data obtained from the factorial experiments were submitted to statistical analysis at 90% confidence level, for fitting to the second order polynomial equation. The ANOVA results, including the regression coefficients for the coded polynomial equation, the determination coefficients (R^2^) and *p*-values, are listed in Table 2.

**TABLE 2:** Results for the regression coefficients and analysis of variance (ANOVA).

Parameter	Coefficient	Particle size		PDI	
		Y_1_	*p*-value	Y_2_	*p*-value
Linear	*β*_*0*_	195.69	0.000000	0.38	0.000000
X_1_	*β*_*1*_	−42.47	0.017310	—	—
X_2_	*β*_*2*_	139.74	0.000001	0.13	0.007874
X_3_	*β*_*3*_	−119.10	0.000005	−0.13	0.011642
Quadratic					
X_1_	*β*_*1*_^*2*^	—	—	—	—
X_2_	*β*_*2*_^*2*^	—	—	—	—
X_3_	*β*_*3*_^*2*^	60.57	0.001530	—	—
Interaction					
X_1_X_2_	*β*_*12*_	−61.38	0.010057	—	—
X_1_X_3_	*β*_*13*_	45.38	0.043526	—	—
X_2_X_3_	*β*_*23*_	−72.38	0.003657	—	—
*p*-value		1.72084 × 10^−6^	—	0.00272667	—
R^2^		*0.94*	—	*0.50*	—

^a^ X_1_ = Proportion of RSO, in the oily phase; X_2_ = total concentration of lipids; X_3_ = total concentration of surfactants; R2 = coefficient of determination.

Only significant values were included.

The ANOVA of the response variables resulted in R^2^ of for 0.94 and 0.50, respectively for average size (Y_1_) and PDI (Y_2_), indicating that the models were able to explain 94% and 50% of the variations observed in the experimental data. In addition, both *p*-values for Y_1_ and Y_2_ were lower than 0.1 (Table 2). Thus, this model was considered predictive of average size but not of the PDI values of the formulations with the selected components and under the studied conditions, as this property was only 50% explained by this model. The PDI is an index associated with the degree of homogeneity of a sample. The smaller the value, the greater the homogeneity of the particle diameter distribution in the system (Danaei et al., 2018).

Thus, the statistically significant regression coefficients (90% confidence level) were included in the equation used to analyze the behavior of the adjusted mathematical model for the variable particle size:

$$Y_{1}=195.69-42.47\;X_{1}+139.74\;X_{2}-119.10\;X_{3}+60.57\;X_{3}^{2}-61.38\;X_{1}X_{2}+45.38\;X_{1}X_{3}-72.38\;X_{2}X_{3}$$

$$Y_{2}=0,38+0,13X_{2}-0,13X_{3}$$

where Y represents the dependent variables (Y_1_ = average size, Y_2_ = PDI) and X corresponds to the coded independent variables (X_1_ = proportion of oil, X_2_ = total lipids, X_3_ = total surfactants).

Through the equation, the notable influence of all independent variables on the average size can be discerned. Regarding the linear coefficient, the variable with the greatest impact was observed to be X_2_. As the total lipid concentration increased, particle size also increased. Conversely, variables X_1_ and X_3_ exhibited an inversely proportional relationship; higher proportions of oil and surfactant resulted in smaller particle sizes. Only the variable X_3_ (total surfactants) displayed a significant quadratic coefficient. In terms of the interaction coefficients, it is noteworthy that the interaction between total lipids and total surfactants (X_2_X_3_) was the most influential, confirming that both variables exert a more pronounced influence on the response variable.

Regarding the PDI variable (Y_2_), the equation displayed neither significance nor predictive power. From the equation, it is evident that only the linear variables X_2_ and X_3_ exert an influence on the response variable, leading to the omission of response surface plotting for PDI.

### 3.2 Effect of the NLC-RSO composition on the particle size

**FIGURE 1:**
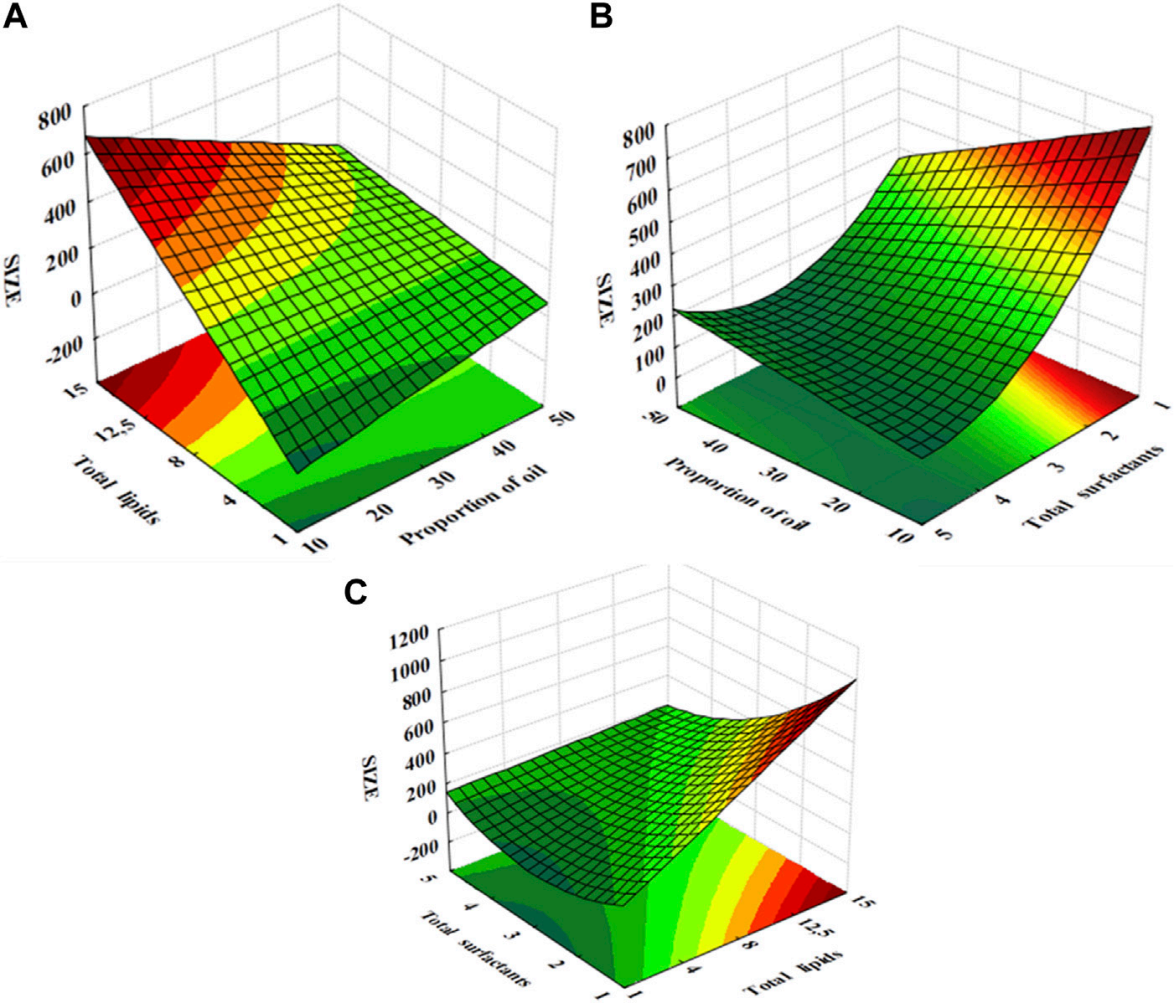
Response surfaces of particle size as a function of **(A)** total lipids and proportion of oil (total surfactants = 3% w/v); **(B)** RSO proportion and total surfactants (total lipids = 8% w/v); **(C)** total surfactants and total lipids (RSO proportion = 30% w/w).

The particle size is usually the first quality parameter to be evaluated in nanoparticle-based products because the composition and preparation conditions directly influence this feature. In addition, the performance of the developed nanoparticles as nanocarrier systems is related to the particle size, shelf life, stability, encapsulation efficiency of the bioactive compound, release profile, biodistribution, and cellular uptake, among other biological properties (Danaei et al., 2018). Thus, the influence of three composition factors was assessed: oil proportion, total lipids, and total surfactants. The response surfaces resulting from the statistical analysis are shown in Figure 1.

The average size of the nanoparticles increased proportionally with an increase in total lipid concentration and a reduction in the proportion of RSO (Figure 1A). Therefore, to obtain smaller nanoparticles, it is important to reduce the total lipid concentration while increasing the proportion of oil to the limit of the desired bioactive content. When the concentration of solid lipids in the formulation increases, the viscosity of the colloidal dispersion also increases, which hampers the emulsification process and results in larger nanoparticles. However, the addition of liquid lipids such as RSO reduces the system viscosity and, consequently, contributes to the size reduction (Veni and Gupta, 2020; Ramadan et al., 2023).

A reduction in the surfactant concentration and oil proportion promoted a significant increase in particle size (Figure 1B). Surfactants are necessary to reduce the interfacial tension so that the molten lipid matrix can be more effectively homogenized with the aqueous phase during preparation, resulting in smaller nanoparticles. In addition, the surfactant layer in the lipid matrix can act as a barrier to the aggregation of nanoparticles in the dispersion, prolonging the long-term stability of the formulation (Zhang et al., 2013; Veni and Gupta, 2020). Finally, increasing the lipid content and reducing the surfactant concentration yielded nanoparticles with larger particle sizes (Figure 1C). Higher surfactant/lipid ratios are important for producing smaller nanoparticles because surfactants should cover the entire surface of the lipid nanoparticles to allow their dispersion in the aqueous phase during the emulsification process (Zhang et al., 2013).

### 3.3 NLC-RSO optimization using the desirability function

Critical quality attributes of the product (response variables) are generally affected by a combination of various input factors in the formulation composition and manufacturing process. Therefore, it is essential to select the best combination of input variable settings for a process such that all response variables can be optimized to reach the desired specifications. Then, the optimization of the NLC-RSO composition was performed using a multi-response method for desirability (Derringer and Suich, 1980; Pelissari et al., 2013; Pal and Gauri, 2018) for simultaneous optimization of the three composition variables. This method involves transforming each response variable (Y_i_) into an individual desirability function (d_i_) ranging from 0 to 1. If the response was outside the pre-established acceptable region, it was defined as di = 0, whereas a fully desirable response (goal) was defined as d_i_ = 1 (Pelissari et al., 2013; Pal and Gauri, 2018).

**FIGURE 2:**
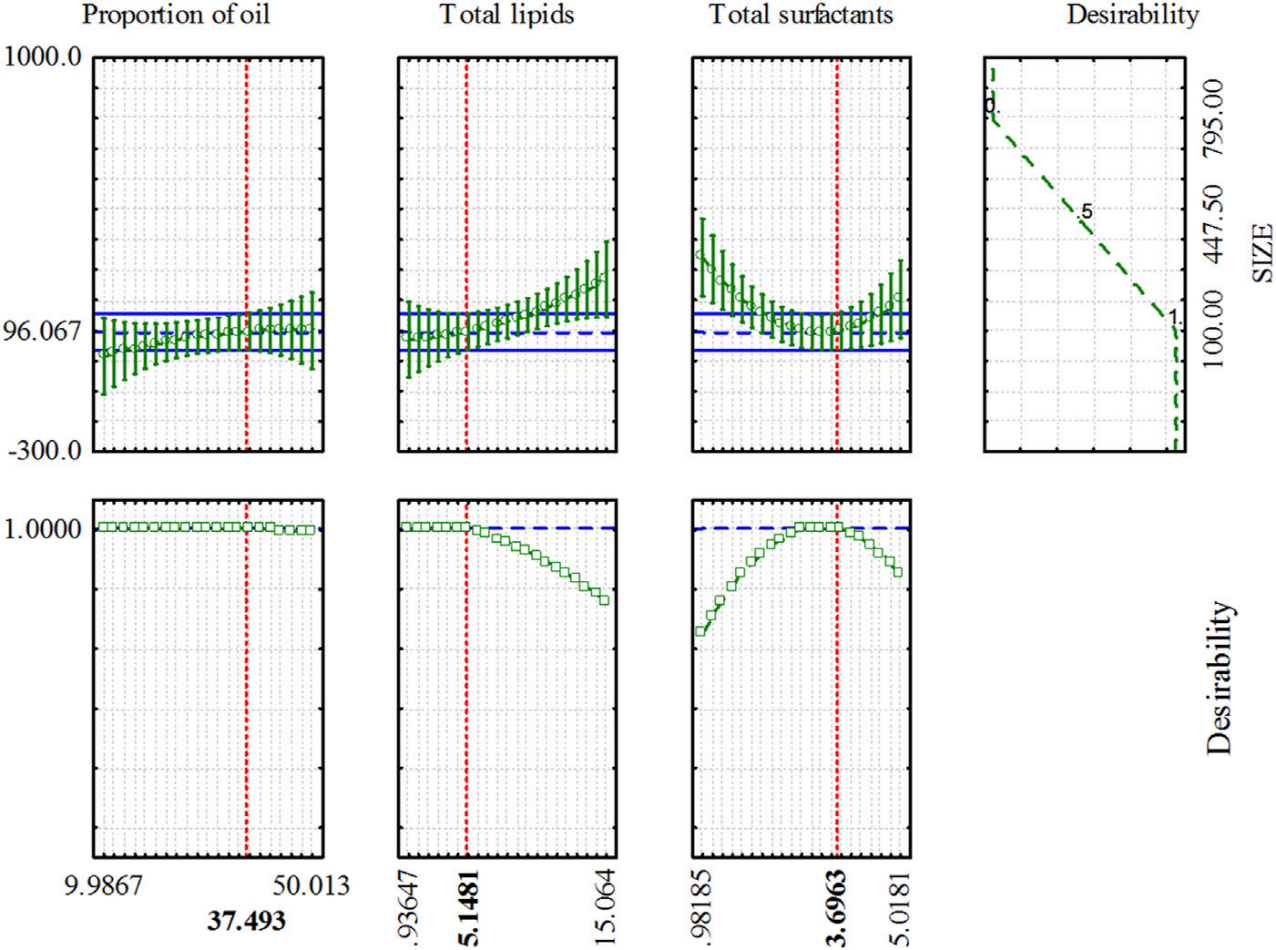
Simultaneous optimization of the NLC-RSO composition as a function of the composition variables, predicted response variables and desirability profiles.

This multi-response optimization of the predicted profiles for the response variables had a mathematical model considered valid for the particle size, as is shown in Figure 2 with its respective desirability function profiles for each of the investigated variables (total lipids, total surfactants, and RSO proportion). Thus, the maximum global desirability (1.00) would be obtained with the optimized composition of the NLC-RSO: oil proportion of 37.49% (w/w), total lipids of 5.15% (w/v), and total surfactant = 3.70% (w/v).

Experimental validation was performed by preparing the optimized NLC-RSO formulations in triplicate. The expected particle size and experimental results were expressed as mean values, and the relative deviation was calculated (Table 3). Based on the relative deviation obtained (−7.6%), the optimization methodology was considered satisfactory. The experimental value obtained for the particle size was less than 100 nm, which favored the biopharmaceutical properties of the nanoparticles, such as absorption, cellular uptake, controlled release, and bioavailability of the encapsulated bioactive metabolite. In addition, nanoparticles smaller than 150 nm can enter or exit blood capillaries in the hepatic endothelium or even in a tumor microenvironment, while nanoparticles between 20–100 nm can be distributed to the bone marrow, spleen, and liver sinusoids, leaving the bloodstream through the capillaries (Danaei et al., 2018).

**TABLE 3:** Experimental validation in the optimized composition conditions of the NLC-RSO.

Property	Predicted value	Experimental value	Relative deviation (%)^a^
Size (nm)	96.0	89.2	−7.6

^a^ Relative deviation = [(experimental value - predicted value)/experimental value] × 100.

Therefore, as the average size obtained with the optimized formulation was lower than 100 nm, the use of this NLC-RSO is promising to deliver this bioactive to the organism (Danaei et al., 2018). The PDI obtained (0.21 ± 0.01) was lower than 0.3, which indicates a monodisperse and stable system.

### 3.4 NLC-RSO characterization

#### 3.4.1 Zeta potential

The ZP of the optimized formulation was highly negative (−33.03 ± 0.5 mV), which may be indicative of good electrostatic stabilization of the nanodispersion. The greater the absolute value of ZP, whether negative or positive, the greater the repulsion between particles with the same charge, circumventing the natural tendency of particle aggregation. Thus, the particles are expected to remain stable in the dispersion with a constant size and the same release profile as the encapsulated content over time (Abosabaa et al., 2021; Dhiman et al., 2021).

#### 3.4.2 FTIR spectroscopy

**FIGURE 3:**
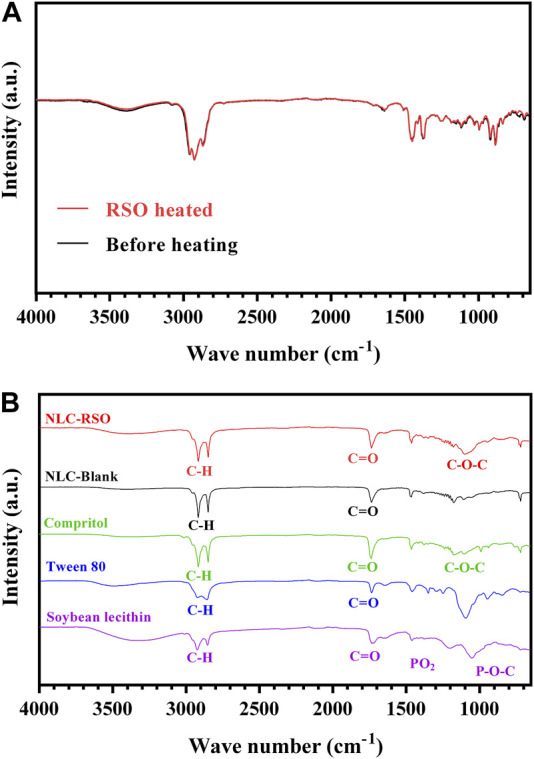
FTIR absorption spectra. **(A)** Overlapped absorption spectra of RSO before and after heating up to 85°C. **(B)** Absorption spectra of the isolated formulation components and NLC-RSO.

FTIR spectra were obtained for each component and lyophilized NLC-RSO formulation (Figure 3). Since the preparation method of NLC-RSO involves heating up to 85°C in the emulsification step, the FTIR spectra of RSO were obtained before and after heating to this temperature. Both spectra were overlapped (Figure 3A), and the correlation coefficient (r = 0.997) obtained was high, indicating that heating did not change the oil characteristics, with no significant changes in the functional groups.

The isolated components showed their characteristic signals in their respective FTIR spectrum (Figure 3B). For Compritol, bands were observed at 2,919 cm^−1^ and 2,850 cm^−1^ (both C–H stretching vibrations), 1740 cm^−1^ (carboxylic acid C=O stretching associated with behenic acid), and between 1,070 and 1,150 cm^−1^ (C–O–C ether stretching), which are related to the ester bonds between the fatty acids and glycerol (Aburahma and Badr-Eldin, 2014). For Tween 80, bands at 2,929 cm^−1^, 2,850 cm^−1^ (both C-H stretching vibrations), and 1740 cm^−1^(C=O stretching of ester groups) (Joshi et al., 2016; Almeida et al., 2022). For soy lecithin, a large band was observed at 3,650–3,100 cm^−1^ (OH elongation) and the three phospholipid bands present in phosphatidylcholine were evidenced between 1,765 and 970 cm^−1^: 1,750–1,720 cm^−1^ (C=O vibration), 1,200–1,140 cm^−1^ (PO_2_ vibration) and 1,140–970 cm^−1^ (P-O-C bonds) (Kuligowski et al., 2008; Almeida et al., 2022).

In the FTIR spectrum of NLC-RSO, bands were observed at 2,920 cm^−1^ and 2,850 cm^−1^ (alkane C–H stretching vibrations), 1740 cm^−1^ (carboxylic acid C=O stretching vibrations), and near 1,470 cm^−1^ and 1,380 cm^−1^ (CH_2_ and CH_3_ vibrations in long hydrocarbon chains) (Hadjiivanov et al., 2021). The NLC-RSO spectrum was similar to that of the corresponding blank NP formulation, with the same absorption band profile. There were also no significant changes compared to Compritol, indicating the absence of incompatibility between the components of the nanoparticles and satisfactory encapsulation of the compounds in the lipid matrix (Filopoulou et al., 2021). This indicated that the oil was satisfactorily incorporated into the lipid matrix and that Compritol and the nanoparticles were different entities.

#### 3.4.3 Powder X-ray diffraction

**FIGURE 4:**
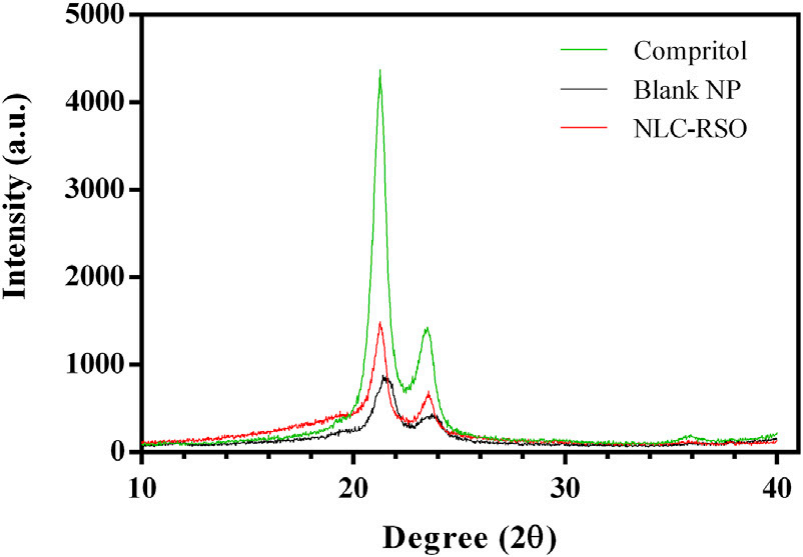
Overlay of the XRD patterns of NLC-RSO, blank NP and Compritol, the main solid component of the lipid matrix.

Powder XRD analysis indicated the crystal lattice arrangements of a solid compound based on the signals present in the resulting diffractograms. These measurements are also useful for determining the subcell parameters and polymorphic forms of glycerides, which are components of lipid nanoparticles. The XRD diffractograms obtained for Compritol, the main solid matrix component, the lyophilized blank NP and NLC-RSO formulations are shown in Figure 4. All diffractograms presented the characteristic short spacings of the β’ form at 21.2° (high intensity) and 23.4° 2θ (lower intensity), corresponding to 0.420 and 0.382 nm (Castro et al., 2008; Almeida et al., 2022). Blank NP (nanoparticles without RSO) showed a pattern similar to that of Compritol, but with slightly broader peaks and lower intensity.

However, NLC-RSO showed broader reflections than Compritol, which is indicative of a less organized lipid matrix. The absence of sharp peaks in this diffractogram indicates the amorphous nature of the NLC-RSO lipid matrix, owing to its composition of solid lipids, oils, and other amorphous substances such as soy lecithin (Shaveta et al., 2020; Chaudhari et al., 2021). The tendency to form a small shoulder at 19° (0.460 nm), observed in both formulations (blank NP and NLC-RSO), indicates the partial formation of the β_i_ intermediate form and the formation of a crystalline lattice of less perfection in the nanoparticles, compared to the pure lipid (Compritol) (Castro et al., 2008; Almeida et al., 2022).

#### 3.4.4 Color parameters

The CIE Lab is an international standard for color measurement developed by the *Commission Internationale de l’Éclairage* (CIE) in 1976, where the colors are described by parameters of lightness (L*), in the range of 0 (black) to 100 (white); the chroma a^*^ (red content: +a^*^, green content: a^*^); and the chroma b^*^ (content yellow: +b^*^, blue content: b^*^) (Andrade et al., 2023; Rezaei et al., 2023). From the color variation (ΔE^*^), it is possible to confirm whether or not there was an effective color change (Vieira et al., 2019; Andrade et al., 2023; Rezaei et al., 2023).

The L^*^ value of the RSO sample was 4.39 ± 0.50, indicating a very close proximity to dark colors, while more lightness (56.62 ± 0.05) was observed for NLC-RSO (*p* < 0.05). The a^*^ values for both RSO and NLC-RSO were observed in the range of green (−0.74 ± 0.05 and −0.86 ± 0.01, respectively), while the b* values were different between them, with blue evidenced for RSO (−0.20 ± 0.08) and yellow, for NLC-SO (5.66 ± 0.02).

The value of ΔE* value of NLC-RSO was higher (56.9) than that of RSO. In addition, this difference was lower, 11.6, and 7.7, compared to Compritol and blank NP (without RSO), respectively. Therefore, the NLC-RSO color was closer to Compritol and the blank formulation than the RSO original color, which was darker than the formulations. In fact, the color of both formulations was predominantly white owing to the presence of Compritol, a white solid lipid, in the matrix. White color or pastel tones are often required for pharmaceutical formulations or food supplements, as they are more acceptable to patients and users. Formulations with these colors are also more easily incorporated into a food matrix with negligible changes to the background color (Aburahma and Badr-Eldin, 2014; Mohana Priya et al., 2020).

#### 3.4.5 Moisture content and wettability

Moisture content represents the total water content of the lyophilized powder. This is an important factor to predict the stability of dried powders, since high water content may cause particle agglomeration, accelerated microbial growth, enhanced lipid oxidation, and consequently, shorter shelf life (Ulloa-Saavedra et al., 2022; Andrade et al., 2023). The low moisture percent found in the lyophilized NLC-RSO powder (2.86% ± 0.00014%), indicates lack of susceptibility to microorganism growth and the effectiveness of the lyophilization process (Alp and Bulantekin, 2021; Xie et al., 2021).

Wettability is the ability of water to remain in contact with the solid surface of the samples and is a direct result of the intermolecular interactions in the solid-liquid interface while they are in contact. The wettability found for NLC-RSO was considered low (>60 min), which is due to the hydrophobic nature of the lipid matrix of the lipid nanoparticles, as in NLC formulations. Coating with surfactants is a mitigating factor; however, wettability is much slower than that observed in a hydrophilic matrix (Kubiak et al., 2011; Duta et al., 2015; Freire et al., 2017).

### 3.5 Characterization of the RSO

#### 3.5.1 RSO metabolites

The chemical composition of RSO was determined by GC-MS and a total of 33 metabolites were identified (Table 4). Four major metabolites were identified in the characterization of RSO: the sesquiterpenoid 5-hydroxy-calamenene (7.84%), the two sesquiterpenes germacrene D (8.24%) and δ-cadinene (11.13%), and the monoterpene linalool (19.71%). 5-hydroxy-calamenene and linalool have already been reported as the most abundant metabolites in previous studies of *Croton sp.* essential oils (Chaves et al., 2006; Souza et al., 2006; Azevedo et al., 2021). 5-hydroxy-calamenene has antimicrobial and antioxidant activities (Azevedo et al., 2013) and linalool has anti-inflammatory, analgesic, hypotensive, vasorelaxant, antinociceptive, and antimicrobial activities (Guo et al., 2021). As σ-cadinene and germacrene D, others sesquiterpenes were identified in RSO: spathulenol (2.43%), γ-muurolene (1.75%) and germacrene B (0.95%), which were also present in the essential oil of *Croton heliotropiifolius*. The abundance of sesquiterpenes is a characteristic of the profile of *Croton sp.* essential oils (Araújo et al., 2017). Thus, such findings are in agreement with the literature, which particularly attributes the anti-inflammatory and antioxidant properties of this oil to linalool and 5-hydroxy-calamenene.

**TABLE 4:** Percentage composition of RSO determined by GC-MS.

Metabolites	AI	(%)
α-thujene	924	0.20
α-pinene	931	1.38
Sabinene	970	3.77
β-pinene	977	0.50
o-cymene	1,023	0.45
D-limonene	1,027	0.49
eucalyptol	1,030	0.34
cis-β-ocimene	1,043	0.36
Linalool	1,100	19.71
4-terpineol	1,178	0.81
α-cubebene	1,344	0.36
cyclosativene	1,364	1.29
α-copaene	1,372	4.59
β-bourbonene	1,379	1.92
β-elemene	1,386	0.30
β-caryophyllene	1,415	6.54
β-copaene	1,425	0.98
α-humulene	1,450	2.41
alloaromadendrene	1,455	4.09
γ-muurolene	1,470	1.75
germacrene D	1,476	8.24
*trans*-muurola-4 (14),5-diene	1,490	1.97
α-muurolene	1,494	1.29
γ-cadinene	1,510	1.45
δ-cadinene	1,514	11.13
germacrene B	1,552	0.95
*E*-nerolidol	1,557	0.37
spathulenol	1,570	2.43
caryophyllene oxide	1,575	1.06
globulol	1,587	1.16
1-epi-cubenol	1,621	0.88
τ-cadinol	1,636	0.81
τ-muurolol	1,649	0.56
5-hydroxycalamenene		7.84
Total		92.38

AI, arithmetic index.

#### 3.5.2 Radical scavenging measurements

**FIGURE 5:**
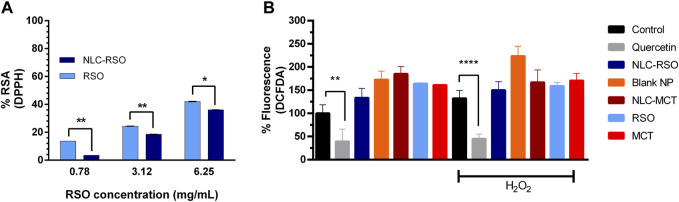
Antioxidant profile of RSO and *in vitro* cell activity **(A)** Comparative DPPH radical scavenging (RS) for RSO and NLC-RSO. **(B)** Intracellular ROS levels measured by DCFDA fluorescence intensity compared to non-treated BEAS-2B cells (control group). The cells were treated with 25 μg/mL NLC-RSO, blank NP, NLC-MCT, RSO or MCT, for 24 h. Quercetin (200 µM), applied for 1 h, was used as positive control. Oxidative stress was induced by using hydrogen peroxide (H_2_O_2_) 50 µM for 1 h (*n* = 4). Data were expressed as mean ± standard deviation (n = 4) (*p < 0.05 **p = 0.0096 ****p < 0.0001).

Although not used in the context of pharmacological assays (Heinrich et al., 2022), RS measurements were also performed as an indirect method to verify the ability of nanoparticles to protect bioactive metabolites. RSO showed considerable DPPH-radical scavenging (Figure 5A) with a dose-dependent response, and the DPPH scavenging varied proportionally with the concentration of RSO used (r = 0.9917). The linear regression analysis to obtain the standard curve for the determination of BHT equivalent showed a high correlation (r = 0.9975), allowing the determination of the BHT-equivalent DPPH scavenging, 7.35 ± 0.28 mg BHT equivalent/g of RSO.

The NLC-RSO were compared with the free oil in the range of 0.78–6.25 mg/mL, and a reduction in DPPH-radical scavenging was observed after the encapsulation in this concentration range. For instance, at 0.78 mg/mL, the DPPH-radical scavenging was reduced from 13.7% (free RSO) to 3.6% (NLC-RSO), indicating bioactive protection after the encapsulation in NLC-RSO. NLC-RSO could not be tested at concentrations above 6.25 mg/mL due to the opacity limitations of the method. Therefore, the lipid nanoparticles partially protected the bioactive oil from direct contact degradation.

### 3.6 Cytotoxicity profile of free RSO and NLC-RSO

The effects of RSO and NLC-RSO on the metabolic activities of A549 and BEAS-2B cells were assessed by measuring resazurin reduction. A549 is a non-small cell lung cancer cell line, considered the principal model used *in vitro* for the study of lung carcinogenesis (Hillyer et al., 2018; Subramaniyan et al., 2022), and BEAS-2B is an immortalized and non-cancerous strain established from normal human bronchial epithelium, used to evaluate the *in vitro* cytotoxicity and potential pulmonary toxicity of drugs and biological agents (Han et al., 2020).

**FIGURE 6:**
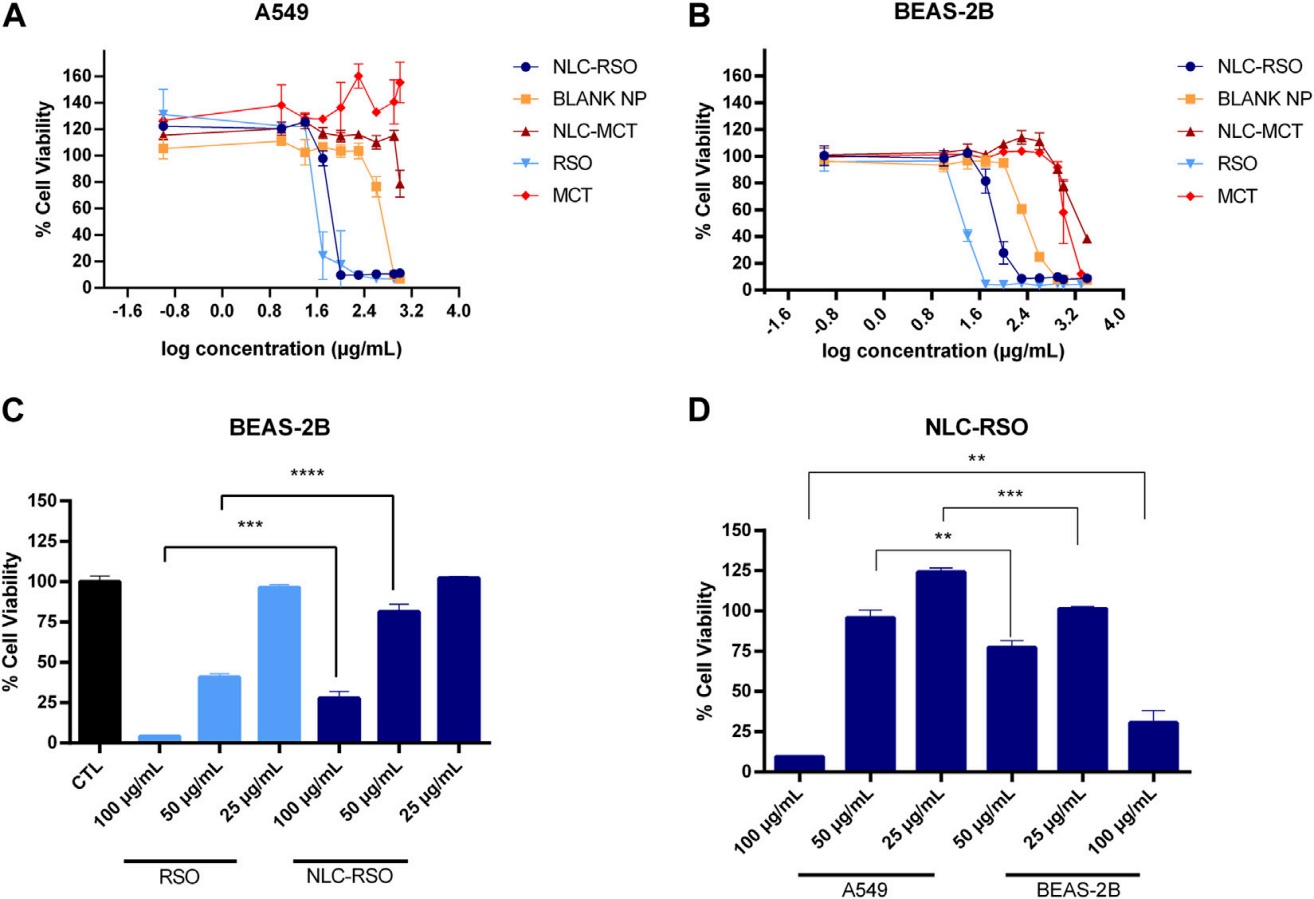
Effect of NLC-RSO, blank NP, NLC-MCT, RSO and MCT on cell viability after 24 h contact. Dose-response curves in **(A)** A549 and **(B)** BEAS-2B cells. **(C)** Comparative effect of RSO and NLC-RSO on decreasing viability in BEAS-2B cells. **(D)** Comparative profile of A549 and BEAS-2B cell viability upon NLC-RSO treatment. Data were expressed as mean ± standard deviation (*n* = 4) (****p < 0.0001, ***p = 0.0003 and **p = 0.0013).

NLC-MCT and blank NP were used as controls to evaluate oil delivery by the nanocarriers. A549 cells (Figure 6A) showed IC_50_ values of 99.59 ± 27.71 μg/mL for NLC-RSO, 59.84 ± 29.30 μg/mL for RSO, and 557.1 ± 21.50 μg/mL for the blank NLC (Table 5). Cell viability was not affected by NLC-MCT and MCT treatments. For BEAS-2B cells (Figure 6B), the IC_50_ was 83.87 ± 14.73 μg/mL for NLC-RSO, 18.91 ± 13.48 μg/mL for RSO, 250 ± 12.20 μg/mL for blank NP, 3,507 ± 12.88 μg/mL for NLC-MCT and 1756 ± 17.35 μg/mL for MCT (Table 5).

**TABLE 5:** Determination of IC_50_ values of NLC-RSO, blank NP and NLC-MCT in different cell lines by resazurin metabolization assay. All values are reported as mean ± standard deviation, in µg/mL. (*n* = 4).

Treatments	IC_50_ (µg/mL)	
	A549	BEAS-2B
NLC-RSO	99.59 ± 27.71	83.87 ± 14.73
Blank NP	557.1 ± 21.50	250 ± 12.20
NLC-MCT	Unstable	3,507 ± 12.88
RSO	59.84 ± 29.30	18.91 ± 13.48
MCT	Unstable	1756 ± 17.35

IC_50_, inhibitory concentration 50%.

NLC-MCT did not affect the A549 cells viability, while BEAS-2B cells showed a reduction in the viability in higher concentrations. The NLC-MCT treatment was used as a control for oil delivery, confirming the effectiveness of NLCs as delivery systems and that the cytotoxic effect from NLC-RSO treatment was only due to the RSO metabolites in the nanoparticles and not from the nanocarrier itself. The same viability profile was observed when the A549 and BEAS-2B cell lines were treated with blank NPs. The cytotoxicity induced by NLC-RSO was similar to that observed for free RSO up to the concentration of 25 μg/mL. However, at the range of 50–100 μg/mL, the free RSO showed higher cytotoxicity than NLC-RSO (Figure 6C). BEAS-2B cells were more susceptible to the cytotoxic effect of NLC-RSO than A549 cells (Figure 6D) at the concentration of 100 μg/mL (*p* = 0.0003). The same profile was observed in the absence of serum supplementation, in which BEAS-2B cells were also more susceptible than A549 cells for NLC-RSO treatment (25 μg/mL) (data not shown). Therefore, the effective delivery of NLC containing the bioactive oil was observed after 24 h when compared to the NLC without bioactives and this process can lead to a reduction in the cellular toxicity (Poovi and Damodharan, 2020; Moura et al., 2021).

### 3.7 Quantification of cellular reactive oxygen species levels

The assessment of a preliminary *in vitro* antioxidant activity of NLC-RSO and RSO was performed by quantifying the levels of reactive oxygen species (ROS) in the BEAS-2B cell line subjected to stress with H_2_O_2_ or not, which were previously treated in a non-cytotoxic concentration of NLC-RSO, blank NP, NLC-MCT, free RSO and free MCT (Figure 5B). At a high but a non-cytotoxic concentration, the nanoparticle- and oil-treated cells did not show alterations in intracellular ROS levels.

Despite RSO have showed DPPH-radical scavenging at concentrations as high as 0.78 mg/mL, when performing the intracellular ROS detection in lower concentrations such as 0.025 mg/mL, there was no decrease in cellular ROS production. Considering the analysis of plant-derived metabolites, it is necessary to use several techniques to assess the antioxidant potential (Ververis et al., 2020). Although the DPPH reaction positively indicated for RSO metabolites able to scavenge the DPPH radical, these concentrations were markedly cytotoxic, whereas a non-cytotoxic dose could not reduce cellular ROS levels. Other substances can act as antioxidants under certain conditions and also have cytotoxic and pro-oxidant effects in other contexts (Thá et al., 2021). For instance, while the essential oil of *Origanum onites* and carvacrol exhibit free radical scavenging, the reduction in intracellular ROS generation was not statistically significant following the application of the essential oil and p-cymene in HCT116 and HepG2 cells (Becer et al., 2022).

## 4 Conclusion

The optimized NLC-RSO formulation was produced through a 2^3^ factorial design and its characterization showed that RSO was efficiently loaded into NLC with a reduced size to deliver its bioactive metabolites to cells. Zeta potential was highly negative and electrostatically stable nanoparticles were produced. FTIR and DRX indicated the formation of RSO-loaded nanoparticles. The color parameter, moisture content and wettability were considered suitable for the intended applications. The major metabolites found in RSO were the sesquiterpenoid 5-hydroxy-calamenene, the two sesquiterpenes germacrene D and δ-cadinene, and the monoterpene linalool. These metabolites are associated with the antioxidant potential and low cellular cytotoxicity of RSO. NLC-RSO were slightly less cytotoxic than free RSO in both evaluated human cell lines. The more aggressive cell line (A549) was more resistant to RSO than BEAS-2B, indicating a possible resistance mechanism in these cells. RSO was observed to have low cellular antioxidant effect and loading the oil into the nanoparticle matrix was important to protect its properties and to perform its cell delivery. The optimized formulation of NLC-RSO offers a promising approach as a potential RSO delivery system that may overcome the limitations of the administration of the free vegetable oil for applications related to cancer treatment, including lung cancer.

## Data Availability

The original contributions presented in the study are included in the article/Supplementary material, further inquiries can be directed to the corresponding author.
